# Follicular regulatory T cells can be specific for the immunizing antigen and derive from naive T cells

**DOI:** 10.1038/ncomms10579

**Published:** 2016-01-28

**Authors:** Meryem Aloulou, Edward J. Carr, Mylène Gador, Alexandre Bignon, Roland S. Liblau, Nicolas Fazilleau, Michelle A. Linterman

**Affiliations:** 1 Centre de Physiopathologie de Toulouse Purpan, Institut National de la Santé et de la Recherche Médicale, U1043, Toulouse F-31300, France; 2 Centre National de la Recherche Scientifique, U5282, Toulouse F-31300, France; 3 Université de Toulouse, Université Paul Sabatier, Toulouse F-31300, France; 4 Laboratory of Lymphocyte Signalling and Development, The Babraham Institute, Babraham Research Campus, Cambridge CB22 3AT, UK

## Abstract

T follicular regulatory (Tfr) cells are a subset of Foxp3^+^ regulatory T (Treg) cells that form in response to immunization or infection, which localize to the germinal centre where they control the magnitude of the response. Despite an increased interest in the role of Tfr cells in humoral immunity, many fundamental aspects of their biology remain unknown, including whether they recognize self- or foreign antigen. Here we show that Tfr cells can be specific for the immunizing antigen, irrespective of whether it is a self- or foreign antigen. We show that, in addition to developing from thymic derived Treg cells, Tfr cells can also arise from Foxp3^−^ precursors in a PD-L1-dependent manner, if the adjuvant used is one that supports T-cell plasticity. These findings have important implications for Tfr cell biology and for improving vaccine efficacy by formulating vaccines that modify the Tfr:Tfh cell ratio.

## Abstract (web summary)

T follicular regulatory cells control the magnitude of the germinal centre response. Here the authors show that these cells display specificity to self as well as foreign antigens, and can arise from Foxp3-negative precursors at early stages of immunization in a PD-L1 dependent manner.

## Graphical abstract

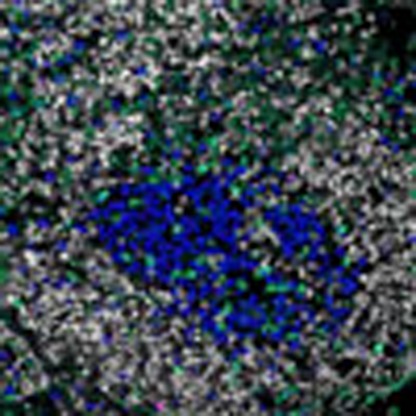

After immunization with T-dependent antigens (Ags), germinal centres (GC) form in secondary lymphoid tissues. GCs are clusters of rapidly dividing B cells that have point mutations introduced into the Ag-binding regions of their B-cell receptor genes by the process of somatic hypermutation. The mutated B cells are then subjected to selection, and often further rounds of mutation, before exiting the GC as long-lived plasma cells or memory B cells. This process is dependent on ‘help' delivered from T follicular helper (Tfh) cells, a specialized subset of CD4^+^ T cells^1^,^2^. Because of the random nature of somatic hypermutation, stringent control of the GC is required to ensure the generation of high-affinity effector cells that do not react with self-Ags^3^. The size and specificity of the GC is influenced by a number of factors, including a subset of suppressive Foxp3^+^ T follicular regulatory cells, coined Tfr cells^4^.

Tfr cells were first identified in the GC of human tonsils^5^ and their biology was elucidated in mice^6^,^7^,^8^. These cells are thought to form after vaccination when Foxp3^+^ precursors co-opt the Tfh cell differentiation pathway, acquiring a Tfh-like phenotype that includes expression of Bcl-6, CXCR5, PD-1 and ICOS. Although Tfr cells share some features of Tfh cells, Tfr cells do not express the B-cell helper molecules interleukin (IL)-21, IL-4 and CD40L that are characteristic of Tfh cells. By contrast, in addition to Foxp3, Tfr cells express a range of proteins that are typical of regulatory T (Treg) cells, such as GITR, Blimp-1 and CTLA-4 (refs 6, 7, 8). Control of Tfr cell differentiation utilizes molecular pathways that are both common to, and distinct from, Tfh cells, including the expression of Helix–Loop–Helix proteins Id2 and Id3 to limit Tfr cell formation^9^ and NFAT to facilitate CXCR5 upregulation on Foxp3^+^ T cells^10^, a function of Ascl-2 in Tfh cells^11^. This change in chemokine receptor expression allows Tfr cells to migrate into the B-cell follicle where they act as suppressor cells within the GC. Tfr cells control the magnitude of the GC response after immunization through molecules such as CTLA-4 (refs 12, 13). They have also been implicated in the control of humoral autoimmunity in mice^6^,^7^,^8^,^10^,^14^.

One of the key unknowns of Tfr cell biology is the Ag specificity of these cells. It is clear that Tfr cells have common features with Tfh cells that are specific for the immunizing Ag^15^,^16^, but also with Treg cells, a T-cell population that has a T-cell receptor (TCR) repertoire skewed towards recognition of self-Ags^17^,^18^,^19^. The observation that Tfr cells derive from Foxp3^+^ precursors and that Tfr cells do not arise from TCR-transgenic CD4^+^ T cells specific for an immunizing Ag^6^,^7^,^8^ prompted the hypothesis that Tfr cells are specific for self-Ag.

Here, we examined the Ag specificity of Tfr cells using peptide:MHC (major histocompatibility complex) class II (pMHCII) tetramers for both self and foreign Ag after immunization. Our results show that Tfr cells are specific for the immunizing Ag, irrespective of whether it is self or foreign Ag. To our surprise, this research also revealed that Tfr cells can derive from Treg cells that are induced in the periphery (pTreg) in addition to thymic derived Treg cells (tTreg), a process that required PD-L1 signalling.

## Results

### Tfr and Tfh cells are specific for the immunizing Ag

**Figure 1:**
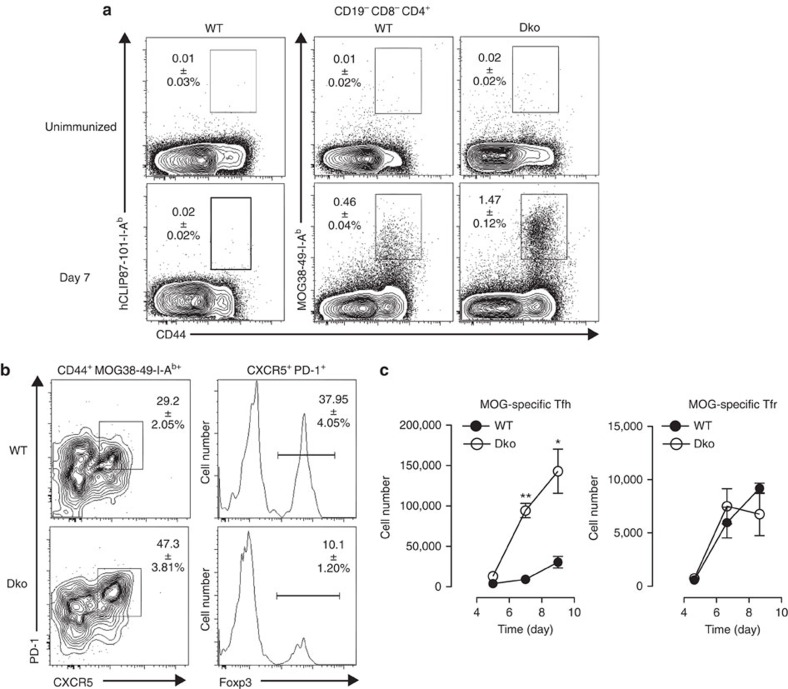
Tfr and Tfh cells are specific for the immunizing Ag. (**a**) hCLIP87-101-I-A^b^, MOG38-49-I-A^b^ and Foxp3 staining of CD19^−^CD8α^−^CD4^+^ dLN (inguinal and periaortic) cells (see Supplementary Fig. 1) from unimmunized WT and Dko mice (top panels) and from WT and Dko mice 7 days after immunization with MOG in CFA (bottom panels). (**b**) WT and Dko mice were immunized with MOG35-55 emulsified in CFA. dLNs were harvested 7 days after immunization for the detection of MOG-specific activated CD4^+^ T cells (CD44^+^ MOG38-49-I-A^b+^) and, among them, the follicular cells (CXCR5^+^ PD-1^+^). Within the latter, we identified Tfr cells (Foxp3^+^) in both WT and Dko mice. (**c**) The mean absolute numbers of total MOG-specific Tfh cells and MOG-specific Tfr cells over time in dLN after immunization of WT (solid circle) or Dko mice (open circle); error bars represent s.e.m. In **a**,**b** the numbers in the dot plots represent the mean±s.e.m. Data shown are from a single experiment with five mice per time point and are representative of three independent experiments. Data were analysed using the non-parametric Mann–Whitney test. *P<0.05; **P<0.01.

Since the TCR repertoire of Tfr cells could be largely skewed towards self-Ag, we took advantage of two different tools to formally investigate Ag specificity of Tfr cells after immunization. The first, pMHCII tetramers, which allows the detection of CD4^+^ T cells specific for the immunodominant peptide (MOG35-55) of the self-Ag myelin oligodendrocyte glycoprotein (MOG) in the context of I-A^b^ in wild-type (WT) C57BL/6 mice. The second, *nefm*^−/−^*mog*^−/−^ mouse model (referred to here as Dko) deficient for the self-Ag MOG and the neuronal cytoskeletal neurofilament-M that thus provides an experimental system in which MOG35-55 can be considered a foreign Ag^20^. With no immunization, we found almost no pMHCII tetramer^+^ CD4^+^ T cells, either Foxp3^−^ or Foxp3^+^, in the inguinal and periaortic lymph nodes (LNs) of both WT and Dko mice (Fig. 1a and Supplementary Fig. 1); this is consistent with the findings of Jenkins and colleagues who demonstrated that there are ∼250 tetramer-binding CD4^+^ T cells per mouse in the steady state^21^,^22^. We then examined this I-A^b^-restricted murine T-cell response in the draining LN (dLN) after subcutaneous (s.c.) immunization with MOG35-55 emulsified in Complete Freund's Adjuvant (CFA). Using an irrelevant pMHCII tetramer (the I-A^b^ human CLIP 87-101), no tetramer^+^ cells were detected in unimmunized and 1W1K-immunized mice (Fig. 1a). In contrast, we detected a population of CD44^+^ and MOG35-55-I-A^b^ tetramer^+^ MOG-specific CD4^+^ T cells in the dLN of both WT and Dko mice 7 days post immunization with MOG35-55 in CFA (Fig. 1a). As expected, in WT mice having MOG as a self-Ag, the frequency of MOG-specific CD4^+^ T cells was reduced compared with Dko mice having MOG35-55 as a foreign Ag (Fig. 1a, 1.47±0.12% versus 0.46±0.04%, *P*<0.05). Likewise, the number of Ag-specific Tfh cells (CD4^+^ CD44^+^ MOG38-49-I-A^b+^ CXCR5^+^ PD-1^+^ Foxp3^−^) was reduced in frequency and in total cell number in WT mice compared with Dko mice (Fig. 1b,c). On the basis of previous observations that Tfr cells derive from Treg precursors^6^,^7^,^8^, we hypothesized that Tfr cells would be specific for self-Ag. Surprisingly, both WT and Dko mice have comparable numbers of Tfr cells (CD4^+^ CD44^+^ MOG38-49-I-A^b+^ CXCR5^+^ PD-1^+^ Foxp3^+^) that are specific for MOG35-55, the immunizing Ag (Fig. 1c). Likewise, the frequency of MOG35-55-specific T cells (MOG38-49-I-A^b+^) among total Tfr cells (CD4^+^ CXCR5^+^ PD-1^+^ Foxp3^+^) was similar in WT and Dko mice (Supplementary Fig. 2). Strikingly, the Tfh:Tfr ratios are higher when specific for non-self-Ag (Dko mice) as compared with self-Ag. This suggests that self-Ag preferentially induces Tfr cells over Tfh cells. Together, these results demonstrate that Tfr cells form in response to the immunizing Ag and can share Ag specificity with their Tfh cell counterparts.

### Tfr cells can derive from pTreg cells

This result raised the possibility that Tfr cells may arise from Treg cells that are induced in the periphery (pTreg) in response to immunization. Previous work suggested that Tfr cells arise from Foxp3^+^ precursors. Indeed, when naive T cells and CXCR5^−^ Foxp3^+^ T cells were co-transferred into T-cell-deficient mice the majority of Tfr cells derive from Foxp3^+^ precursors after immunization^7^,^8^. Moreover, depletion of Treg cells at the time of immunization results in the lack of Tfr cells after the GC has formed, despite the recovery of total Treg cell numbers at this time point^6^, suggesting that Tfr differentiation is initiated at the time of the T-cell priming. Notably, the majority of previous studies on Tfr cells used one of three adjuvants, sheep red blood cells, alum and CFA that are not reported to support pTreg cell generation after immunization. By contrast, Incomplete Freund's Adjuvant (IFA) facilitates the polarization of naive CD4^+^ T cells to Foxp3^+^ pTreg cells^23^. We included IFA in our analyses along with CFA to determine whether an adjuvant that can support pTreg generation has an impact on Tfr cell production.

**Figure 2:**
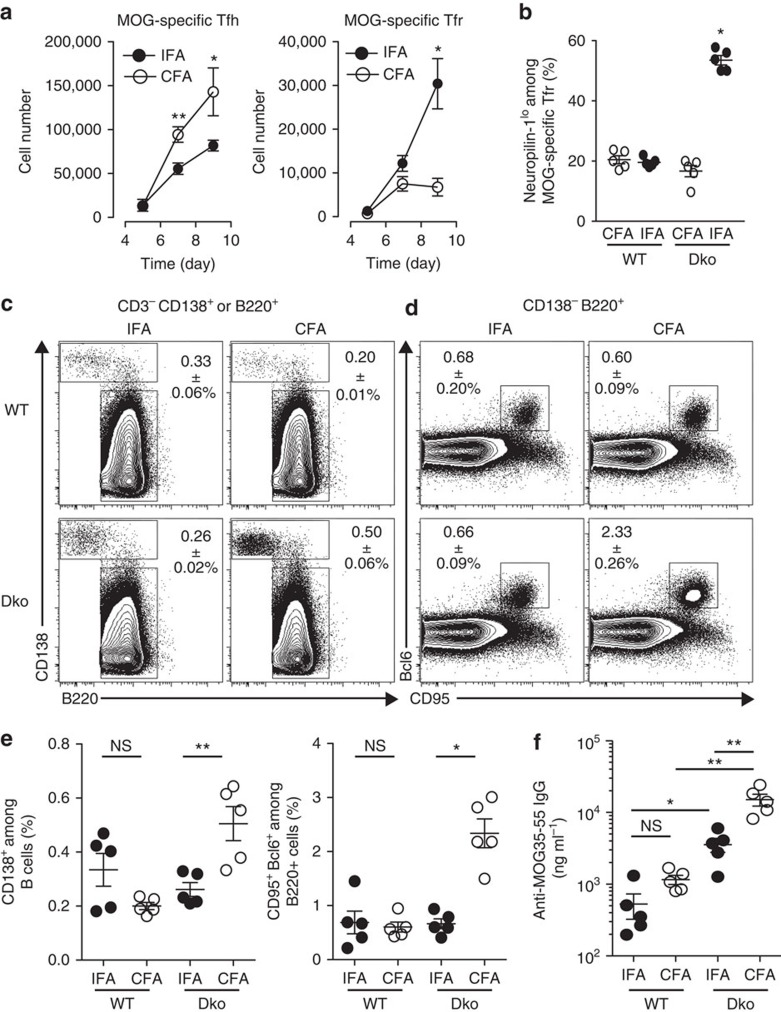
Immunization with MOG in IFA induces increased numbers of Tfr cells that are Neuropilin-1^lo^. Dko mice were immunized with MOG35-55 emulsified in CFA or IFA. (**a**) Absolute numbers of total MOG-specific Tfh cells and Tfr cells of Dko mice immunized with CFA (open circle, as in Fig. 1c) or IFA (solid circle) at indicated time in dLN. (**b**) Neuropilin-1^lo^ expression among MOG-specific Tfr cells 7 days after immunization of WT and Dko mice with MOG35-55 emulsified in CFA (white circles) or IFA (black circles). Data shown are from a single experiment (five mice per group) and are representative of three independent experiments. (**c**) CD138 and B220 staining of CD3^−^ CD138^+^ or B220^+^ dLN cells (see Supplementary Fig. 4) from WT and Dko mice 12 days after immunization with MOG in IFA or CFA. (**d**) CD95 and Bcl6 staining of CD138^−^ B220^+^ cells. (**e**) Frequencies of total plasma cells and GC B cells of WT and Dko mice immunized with IFA (solid circle) and CFA (open circle) in dLN 12 days after MOG immunization. (**f**) Quantity of MOG-specific Ig in the sera of immunized WT and Dko mice estimated using ELISA 12 days after MOG immunization. Data shown are from a single experiment with five mice per group and are representative of two independent experiments. Data were analysed using the non-parametric Mann–Whitney test. ns, not significant, *P<0.05, **P<0.01, mean±s.e.m.

When WT mice were immunized with MOG35-55 emulsified in either IFA or CFA, the proportion and absolute numbers of MOG-specific Tfr and Tfh cells were similar on days 5, 7 and 9 after immunization. In contrast, the number of MOG-specific Tfh cells was significantly higher in CFA-immunized Dko mice than in IFA-immunized Dko mice on days 7 and 9 after immunization (Fig. 2a). Conversely, the absolute number of MOG-specific Tfr cells was higher in IFA-immunized Dko mice on day 9 post immunization when compared with CFA-immunized mice (Fig. 2a). Likewise, the frequency of MOG35-55-specific Foxp3^+^ T cells (MOG38-49-I-Ab^+^ Foxp3^+^) among follicular T cells (CD4^+^ CXCR5^+^ PD-1^+^) was significantly higher in IFA-immunized Dko mice than in CFA-immunized Dko mice (Supplementary Fig. 3). Together, this series of data suggests that IFA supports Tfr cell development, possibly through the induction of pTreg cells. To examine this in more detail, we assessed the expression of Neuropilin-1, a cell surface receptor that is present on tTreg cells and a small number of pTreg cells generated in highly inflammatory environments^24^,^25^. Thus, it is thought that all the Neuropilin-1^lo^ Foxp3^+^ Treg cells are pTreg cells. We found that the increase in total MOG-specific Tfr cells following immunization in IFA correlated with an increase in the Neuropilin-1^lo^ Tfr cell population (Fig. 2b). This only occurred when MOG was a foreign Ag, and not when MOG was a self-Ag (Fig. 2b). These data support the concept that, when MOG is a foreign Ag, MOG-specific Tfr cells can originate from induced pTreg cells. We tested whether higher induction of Tfr cells after IFA immunization as compared with CFA immunization correlates with a difference in the B-cell response. WT and Dko mice were immunized with MOG35-55 emulsified in either IFA or CFA. Twelve days post immunization, we collected dLN and found that the frequencies of total plasma cells (CD138^+^) and of GC B cells (CD95^+^ Bcl6^+^) were similar in WT mice after IFA or CFA immunization (Fig. 2c,d and Supplementary Fig. 4). Similarly, the quantities of MOG-specific IgG in the sera of immunized WT mice were similar after IFA or CFA immunization (Fig. 2e). In contrast, plasma cell frequency, the GC B-cell frequency and the MOG-specific IgG response were all higher in Dko mice immunized with CFA than with IFA (Fig. 2c–e). These data show that there is a correlation between increased number of Tfr cells and a reduced B-cell response. However, these experiments do not exclude a differential effect of IFA and CFA on other cell types that participate in the GC response.

**Figure 3:**
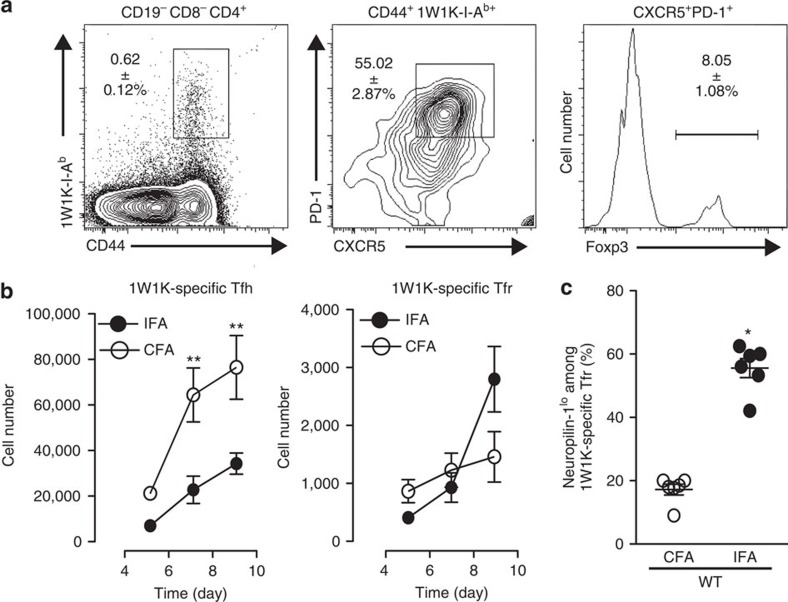
Immunization with a non-self Ag in IFA induces increased numbers of Tfr. (**a**) WT mice immunized with 1W1K emulsified in CFA or IFA. dLNs were harvested for the detection of 1W1K-specific CD4^+^ T cells (CD44^+^ 1W1K-I-A^b+^) and follicular cells (CXCR5^+^ PD-1^+^). Tfr cells (1W1K-I-A^b+^ CXCR5^+^ PD-1^+^ Foxp3^+^) were identified within total 1W1K-specific follicular cells. (**b**) Absolute numbers of total 1W1K-specific Tfh cells and Tfr cells over time in dLN after immunization with CFA (open circle) or IFA (solid circle). (**c**) Neuropilin-1^lo^ expression among 1W1K-specific Tfr cells 7 days after immunization with CFA (white circles) or IFA (black circles). Data are from one experiment of six mice per strain and are representative of three independent experiments. Data were analysed using the non-parametric Mann–Whitney test *P<0.05, **P<0.01, mean±s.e.m.

To confirm that our observations were not only a feature of the MOG-specific CD4^+^ T-cell response in the Dko strain, we extended our studies to another I-A^b^-restricted epitope, the foreign Ag and peptide variant (EAWGALANKAVDKA, called 1W1K peptide hereafter) of the I-E alpha chain (Ea) in C57BL/6 mice^26^. We followed the 1W1K-specific CD4^+^ T cells using the corresponding pMHCII tetramer in the dLN after immunization with 1W1K emulsified either in IFA or CFA. We found almost no 1W1K-I-A^b^ tetramer^+^ CD4^+^ T cells in the inguinal and periaortic LNs of unimmunized WT mice (Supplementary Fig. 5). In contrast, we were able to detect 1W1K-specific Tfh cells (CD4^+^ CD44^+^ 1W1K-IA^b+^ CXCR5^+^ PD-1^+^ Foxp3^−^) and Tfr cells (CD4^+^ CD44^+^ 1W1K-IA^b+^ CXCR5^+^ PD-1^+^ Foxp3^+^) in immunized WT mice (Fig. 3a). The number of 1W1K-specific Tfh cells was decreased in IFA-immunized WT mice as compared with CFA-immunized mice, and more 1W1K-specific Tfr cells 9 days post immunization were found in IFA-immunized mice (Fig. 3b). Likewise, the frequency of 1W1K-specific cells (1W1K-IAb^+^) among total Tfr cells was higher in IFA-immunized WT mice as compared with CFA-immunized mice (Supplementary Fig. 6). Moreover, as observed for MOG-specific Tfr cells in IFA-immunized Dko mice, a higher proportion of 1W1K-specific Tfr cells were Neuropilin-1^lo^ in IFA-immunized mice (Fig. 3c). Together, these data support the hypothesis that Tfr cells specific for foreign Ag can derive from Foxp3^−^ precursors when immunization is performed with an adjuvant that supports this type of T-cell plasticity.

**Figure 4:**
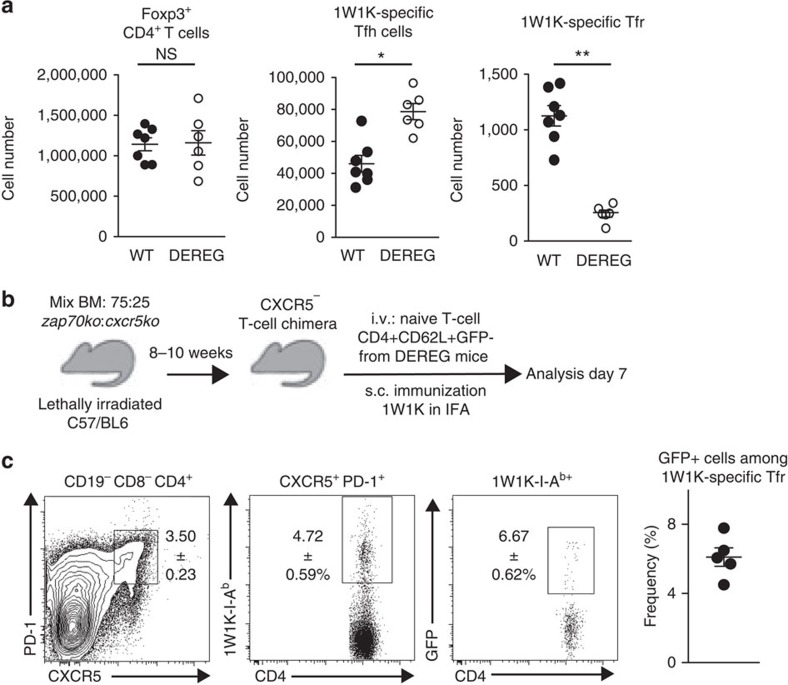
Naive CD4^+^ T cells can become Tfr cells. DEREG and WT mice were immunized with 1W1K emulsified in IFA and were treated with DTx on days 0 and +1. (**a**) Absolute numbers of total Foxp3^+^ CD4^+^ T cells, 1W1K-specific Tfh cells and 1W1K-specific Tfr cells in dLN 7 days after immunization in WT (white circles) and DEREG mice (black circles). Data are representative of six mice per strain. Data were analysed using the non-parametric Mann–Whitney test *P<0.05, **P<0.01, mean±s.e.m. (**b**) Experimental outline. (**c**) Flow cytometric dot plots showing follicular T cells (CXCR5^+^ PD-1^+^), 1W1K-specific follicular cells (1W1K-IA^b+^) and 1W1K-specific Tfr cells (GFP^+^) in the dLN 7 days post immunization with 1W1K in IFA. Data shown are from one experiment with five mice and are representative of two independent experiments.

Previous work suggested that Tfr cells derive from Foxp3^+^ precursors by using a depletion system to remove total Foxp3^+^ Treg cells at the time of immunization. In this system, Tfr cell formation was impaired, suggesting that these cells derive from Foxp3^+^ precursors^6^. However, in light of the data presented here, an alternate interpretation is that Tfr cells may also derive from pTreg cells induced within the first 48 h following vaccination. To test this, we took advantage of the DEREG mouse model in which green fluorescent protein (GFP) and diphtheria toxin receptor expression is under the control of the Foxp3 promoter, the transcriptional regulator of Treg cells^27^. These mice were immunized with 1W1K emulsified in IFA and were treated on the day of immunization and 1 day later with diphtheria toxin (DTx), which because of its short half-life *in vivo* caused the death of tTreg cells and emerging pTreg cells induced within the first 2 days following immunization. Three and five days after immunization, we found statistically significant differences in the total number of Foxp3^+^ CD4^+^ T cells in the dLN of the DTx-treated DEREG and WT mice, and conclude that the Treg cell pool after DTx has not recovered completely at these time points (Supplementary Fig. 7). Seven days after immunization, we found no difference in the total number of Foxp3^+^ CD4^+^ T cells in the dLN of the DTx-treated DEREG and WT mice (Fig. 4a), demonstrating that the Treg cell population has recovered numerically by this time point. We observed an increase in 1W1K-specific Tfh cells in DTx-treated DEREG as compared with DTx-treated WT mice on day 7 after immunization (Fig. 4a), while this was not seen on day 5 after immunization (Supplementary Fig. 7), suggesting that dysregulation of Ag-specific Tfh cells occurs cumulatively over time in the absence of Treg cells. We hypothesized that this may correlate with reduced Ag-specific Tfr cell number. Indeed, very few 1W1K-specific Tfr cells could be detected in DTx-treated DEREG mice compared with DTx-treated WT mice on day 5 (Supplementary Fig. 7) and day 7 after immunization (Fig. 4a). These data support the hypothesis that Tfr cell differentiation begins within the first 48 h after immunization, from either tTreg or pTreg cells, and that loss of Treg cells, at the time of immunization, results in an expansion of the Ag-specific Tfh cell pool.

### Naïve CD4^+^ T cells can become Tfr cells

To determine whether Tfr cells can indeed derive from naive CD4^+^ T cells, we developed mixed bone marrow (BM) chimeras in which all T cells lack CXCR5 expression, into which we could introduce and track naive CD4^+^ T cells from DEREG mice (Fig. 4b). These CXCR5^−^ T chimeric mice were generated by reconstituting irradiated mice with a 75:25% mix of BM from *Zap70*^−/−^ mice, which do not generate peripheral T cells, and from *Cxcr5*^−/−^ mice. In these chimeras, all T cells lack CXCR5 and therefore have a competitive disadvantage for accessing the follicular niche compared with the CXCR5-sufficient CD4^+^ T cells introduced from DEREG mice^28^. This system has the advantage of transferring cells with a competitive advantage over endogenous cells while not introducing cells into lymphopenic hosts. We isolated naive CD4^+^ T cells devoid of Foxp3^+^ cells from DEREG mice (CD19^−^ CD8^−^ CD4^+^ CD44^lo^ CD62L^+^ GFP^−^; Supplementary Fig. 8). These Foxp3^−^ cells were transferred intravenously into the CXCR5^−^ T chimeras, 8 weeks after reconstitution, followed by immunization with 1W1K emulsified in IFA. Seven days later, dLNs were collected and the 1W1K-specific CD4^+^ T-cell response assessed. Follicular T cells (CD4^+^ CXCR5^+^ PD-1^+^) can be found in CXCR5^−^ T chimeras, after transfer of naive CD4^+^ T cells (Fig. 4c). Among these follicular T cells, ∼5% were specific for 1W1K. Strikingly, GFP^+^ cells were detected among the 1W1K-specific CD4^+^ T cells of follicular phenotype (Fig. 4c). As GFP marks Foxp3 expression only in the cells of DEREG mouse origin (that were GFP^−^ at the time of transfer), this demonstrates that Tfr cells can arise from naive Foxp3^−^ precursors after IFA immunization. Together, these data demonstrate that Ag-specific Tfr cells can derive from naive CD4^+^ T cells that are induced to express Foxp3 after immunization.

### PD-L1 promotes the formation of Tfr cells

During T-cell priming, the interaction between programmed death 1 (PD-1) on activated T cells and PD-1 ligand (PD-L1) expressed on the Ag-presenting cells can promote the development and maintenance of pTreg from naive CD4^+^ T cells^29^. Given that PD-1 is expressed highly by Tfr cells^30^ and that Tfr cells are induced within the first 48 h after immunization (Fig. 4a), PD-L1 signalling could be implicated in initiating Tfr cell generation from Foxp3^−^ precursors.

**Figure 5:**
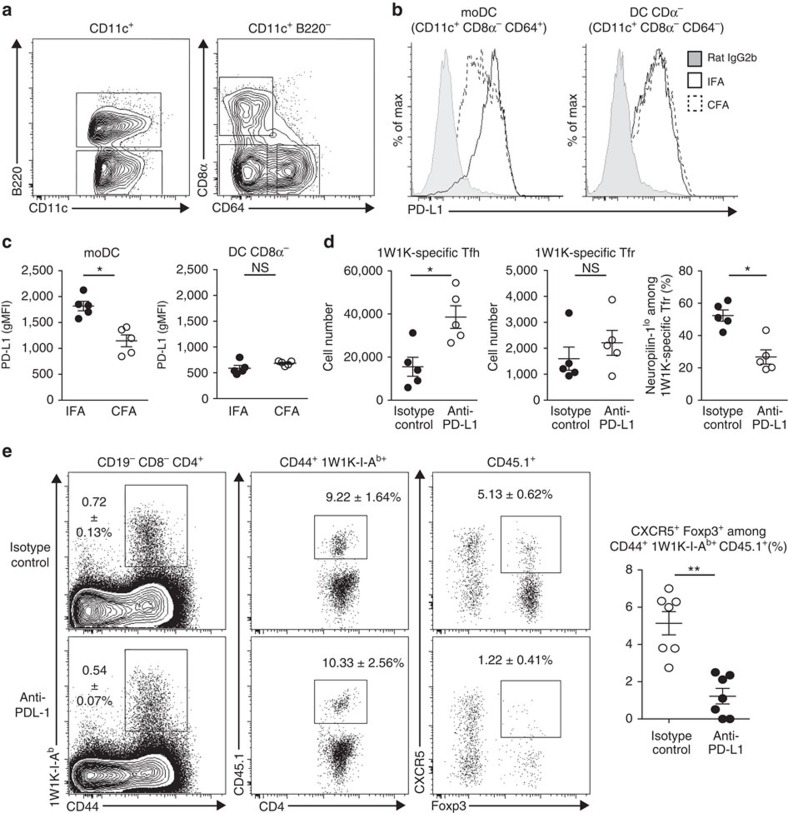
PD-L1 regulates the formation of induced Tfr cells. (**a**) Flow cytometric contour plots showing DC in the dLN 48 h post immunization of WT mice with Ea-OVA in IFA or CFA. (**b**) Histograms showing PD-L1 expression at the surface of conventional CD8α^−^ DC and moDC. (**c**) Geometric mean fluorescence intensity of PD-L1 expression on the surface of conventional CD8α^−^ DC and moDC. Data are from one experiment of five mice per group and are representative of three independent experiments. (**d**) Number of total 1W1K-specific Tfh and Tfr cells in dLN of WT mice 7 days after immunization with 1W1K emulsified in IFA and treated with anti-PD-L1 (white circles) or isotype control (black circles) on days 0 and +2. Frequency of Neuropilin-1^lo^ among 1W1K-specific Tfr cells. Data are from one experiment of five mice per group and are representative of two independent experiments. (**e**) Flow cytometric dot plots showing 1W1K-specific CD4^+^ T cells (CD44^+^ 1W1K-IA^b+^), transferred cells among 1W1K-specific CD4^+^ T cells (CD45.1^+^) and Tfr cells (CXCR5^+^ Foxp3^+^) in the dLN 7 days post immunization with 1W1K in IFA and treated with anti-PD-L1 (white circles) or isotype control (black circles) on days 0 and +2. Data shown are from one experiment with seven mice and are representative of two independent experiments. Data were analysed using the non-parametric Mann–Whitney test. *P<0.05, mean±s.e.m.

To decipher whether PD-L1 was a mechanism by which Tfr cells can differentiate from Foxp3^−^ precursors after immunization with protein emulsified in IFA, but not CFA, we first assessed PD-L1 expression at the surface of dendritic cells (DCs). After immunization with a protein (immunodominant peptide of I-Ea chain conjugated to ovalbumin) in IFA or CFA, we analysed the different subsets of DC in the dLN. Among the total DCs (CD11c^+^ cells), we found plasmacytoid DC (B220^+^), conventional DCs (either B220^−^ CD8α^+^ or B220^−^ CD8α^−^ CD64^−^) and monocyte-derived DCs (moDCs, B220^−^ CD8α^−^ CD64^+^; Fig. 5a). No differential expression of PD-L1 at the surface of plasmacytoid DC or conventional CD8α^+^ DC could be detected after IFA or CFA immunization at 12, 24 or 48 h after immunization (Supplementary Fig. 9). In contrast, conventional CD8α^−^ DC and moDC expressed high levels of PD-L1 at their surface at each time points (Supplementary Fig. 9 and Fig. 5b). Strikingly, while the expression of PD-L1 was similar at the surface of the conventional CD8α^−^ DCs, we found a significant decrease in the geometric mean fluorescence intensity of PD-L1 expression when comparing moDCs from CFA-immunized versus IFA-immunized mice at 24 and 48 h after immunization (Supplementary Fig. 9 and Fig. 5c). This is striking as we have previously shown that moDCs are the main cells presenting the Ag through MHCII molecules after protein immunization^26^. This result supports the hypothesis that the difference in the number of Tfr cells that are induced after IFA- and CFA-based immunization is PD-L1-mediated.

To formally test this hypothesis, we immunized WT mice with 1W1K in IFA and treated them with an anti-PD-L1-blocking antibody the day of immunization and 2 days later. Seven days after immunization, we found an increased number of 1W1K-specific Tfh cells in the anti-PD-L1-treatment group as compared with isotype control group (Fig. 5d). The numbers of 1W1K-specific Tfr cells were similar in both groups (Fig. 5b), and the frequency of 1W1K-specific T cells (1W1K-IAb^+^) among total Tfr cells (CD4^+^ Foxp3^+^ CXCR5^+^) was similar in isotype-treated versus anti-PD-L1-immunized mice (Supplementary Fig. 10). In contrast, we observed a significant decrease in the frequency of Neuropilin-1^lo^ cells among the 1W1K-specific Tfr cells after anti-PD-L1 treatment (Fig. 5d), suggesting that PD-L1 supports Tfr cell generation from Foxp3^−^ precursors after immunization with IFA.

To confirm this observation, we isolated naive CD4^+^ T cells (CD19^−^ CD8^−^ CD4^+^ CD44^lo^ CD25^−^ CD62L^+^) from CD45.1/CD45.1 mice and checked that this population was devoid of Foxp3^+^ cells (Supplementary Fig. 11). These cells were then transferred intravenously into CXCR5^−^ T chimeras as described above (Fig. 4b), followed by immunization with 1W1K in IFA, and were treated with an anti-PD-L1-blocking antibody or an isotype control antibody the day of immunization and 2 days later. Seven days later, dLNs were collected and the 1W1K-specific CD4^+^ T-cell response assessed. We found that the frequencies of 1W1K-specific CD4^+^ T cells (CD4^+^ CD44^+^ 1W1K-IA^b+^) and, among them, the ones arising from the transferred cells (CD45.1^+^) were similar between anti-PD-L1 or isotype-treated mice (Fig. 5e). Strikingly, some of these CD45.1^+^ cells were CXCR5^+^ Foxp3^+^ in isotype-treated mice, while almost no transferred cells acquired this phenotype in anti-PD-L1-treated mice (Fig. 5e). Induced Tfr expressed higher levels of Bcl6 and PD-1 than their CXCR5^−^ counterparts (Supplementary Fig. 12), consistent with these cells being bona fide Tfr cells. To confirm the presence of these induced Tfr cells in the GC, we performed immunofluorescence studies of the dLN and found that, within GL-7^+^ GCs, CD45.1^+^ cells could be identified, among which 13.60±0.90% were FoxP3^+^ (Supplementary Fig. 13). Together, this demonstrates that naive CD4^+^ T cells in response to Ag immunization can become Tfr cells in a PD-L1-dependent manner and localize to the GC.

## Discussion

In this study we have shown that Tfr cells can be specific for the immunizing Ag, and that the differentiation of Ag-specific Tfr cells is initiated at the time of T-cell priming. Surprisingly, it did not matter whether the immunizing Ag was a self- or foreign Ag. We further demonstrate that Tfr cells can derive from Foxp3^−^ precursors in the context of a stimulus that promotes the conversion of CD4^+^ Foxp3^−^ cells into Foxp3^+^ Treg cells, specifically, one that enhances PD-L1 expression on Ag-presenting cells. Whether these ‘induced' Tfr cells emerge from Tfh cells that acquire Foxp3 expression, or from pTreg cells that acquire the follicular fate through CXCR5 expression, remains unknown and will need further investigation. However, Tfh cells cannot be induced to switch on Foxp3 *in vitro*^8^, suggesting that it is more likely that it is pTreg cells that give rise to Tfr cells.

The Ag specificity of Tfr cells has been an outstanding question since their initial characterization. It was initially proposed that Tfr cells were likely to be specific for self-Ag because they were thought to derive exclusively from tTreg^6^,^7^,^8^. Here we show that a small proportion of total Tfr cells is specific for the immunizing Ag, whether it is a self- or foreign Ag. However, because not all Tfr cells were specific for the immunizing Ag, it suggests that these cells are likely a very heterogeneous population. However, the specificity of the Tfr cells not specific for the immunizing immunodominant peptide remains elusive. It is worth noting that in this study ∼30% Tfh cells were specific for the immunizing Ag as measured using tetramer staining. This suggests that either there are limitations in these reagents for detecting all cells that recognize the peptide present on the tetramer, or the specificity of the Tfh and Tfr cell populations is considerably more diverse than that we currently appreciate.

The most unexpected finding of our study is that we demonstrate that Tfr cells can derive from Foxp3^−^ precursors. By using IFA, an adjuvant that promotes pTreg cell formation, we found that naive CD4^+^ T cells can become Tfr cells and are specific for the immunizing Ag. Generation of Tfr cells from pTreg cells occurred predominantly when the immunizing Ag was foreign. We think that this observation may simply reflect the increased precursor frequency of naive CD4^+^ T cells that are non-self-reactive, as it would be expected that a large majority of self-reactive naive T cells would have been removed by thymic selection.

One other striking observation was the fact that the numbers of Ag-specific Tfr cells were similar after anti-PD-L1 treatment but that we observed a significant decrease in the frequency of Neuropilin-1^lo^ cells among these cells, suggesting that PD-L1 supports Tfr cell generation from Foxp3^−^ precursors after immunization with IFA. Moreover, we observed that the number of Ag-specific Tfh cells was increased in anti-PD-L1 monoclonal antibody-treated mice, and thus in mice with less Neuropilin-1^lo^ Tfr cells. This could indicate that Neuropilin-1^lo^ Tfr cells are more efficient than their Neuropilin-1^hi^ Tfr cell counterparts at controlling the Tfh cell response. Or, alternatively, that inhibiting PD-L1 has a direct effect on Tfh cells themselves, or another cell type that acts to control the number of Tfh cells such as regulatory B cells^31^. This result is in apparent contrast to that of Sharpe and colleagues who have shown that inhibiting PD-1 can expand the size of the Tfr cell population without modifying the Tfh cell pool^30^. However, in this study mice were immunized with protein in CFA; thus, based on the results presented here the number of the Neuropilin-1^lo^ Tfr cells would be expected to be low. Taken together, this suggests that PD-L1 may have differential roles in the induction of Tfr cells depending on whether they arise from tTreg or pTreg cells.

This study also showed differences in the ratio between Ag-specific Tfr and Tfh cells depending on several features. Using the same immunizing Ag (MOG35-55), we demonstrated that the Tfr:Tfh cell ratio was higher when this Ag is a self-Ag (in WT mice) as compared with a foreign Ag (in Dko mice). Moreover, the Tfr:Tfh cell ratio was also dependent on the adjuvant used. We found that IFA had a high Tfr:Tfh ratio compared with CFA, likely because of promoting the peripheral induction of naive CD4^+^ T cells into Tfr cells. Taken with our previous study showing that addition of CpG, a TLR9 agonist, to vaccine adjuvant improves Tfh cell differentiation^26^, and with the recent findings of Rookhuizen and De Franco^32^, showing that TLR9 signalling can reduce Tfr cell number, suggests that different adjuvants could be specifically chosen to alter the Tfr:Tfh cell ratio that forms after vaccination to improve efficacy.

## Methods

### Mice

C57BL/6 mice were purchased from Centre d'Elevage Janvier. C57BL/6 *Nefm*^−/−^*Mog*^−/−^ (Dko), C57BL/6 DEREG (gift from T. Sparwasser), C57BL/6 *Zap70*^−/−^ (gift from N. Taylor), C57BL/6 *Cxcr5*^−/−^ (JAX laboratory, stock number 006659 (ref. 33)), C57BL/6 CD45.1 (gift from S. Guerder) were housed in our animal facility. Females of 8–12 weeks of age were used for experimental procedures. All experiments were performed in accordance with national and European regulations and institutional guidelines. Mouse experimental protocols were approved by the local ethics committee (Regional approval No. 311155523, ethical review No. MP/19/58/06/12) or by the UK Home Office (PPL 80/2625).

### BM chimeras

Mice were γ-irradiated (8.5 Gy (850 rad), ^137^Cs source) the day before intravenous injection of 2 × 10^6^ T-cell-depleted BM cells. BM cells were recovered from the femur and tibia. After red cell lysis using Tris-buffered ammonium chloride, cells were enriched in lineage^negative^ cells using the mouse Lineage Cell Depletion kit (Miltenyi Biotec) according to the manufacturer's instructions^26^.

### Immunization

IFA and CFA were from Sigma-Aldrich, and 1W1K (EAWGALANKAVDKA), MOG 35-55 (MEVGWYRSPFSRVVHLYRNGK) and Ea-OVA (ASFEAQGALANIAVDKA-OVA) from Genecust. Mice were immunized s.c. on each side at the base of the tail with 50 μg of peptide (1W1K or MOG) or 100 μg of Ea-OVA in the indicated adjuvant.

### Antibodies

For *in vivo* treatment, intravenous injections of 100 μg 10F.9G2 monoclonal antibody (anti-PD-L1, BioXcell, SKU: BE0101) or Rat IgG2b isotype control (SKU: BE0090) were performed at days 0 and +2 post immunization.

### Flow cytometry

For population analysis, dLNs (inguinal and periaortic) of immunized mice were removed. For staining of Ag-specific cells, 1W1K-IA^b^ and MOG38-49-IA^b^ tetramers were obtained from the NIH Tetramer core facility. Overall, 1 × 10^8^ cells per ml cells were first stained at room temperature for 120 min with an optimal concentration of each tetramer (0.007 mg ml^−1^ for 1W1K-IA^b^ and 0.036 mg ml^−1^ for MOG38-49-IA^b^ and human CLIP87-101-IA^b^). Then, cells were incubated on ice for 45 min with fluorophore-labelled monoclonal antibodies. The following monoclonal antibodies purchased from BD Biosciences were used: anti-CXCR5 (2G8, dilution 1/50), anti-CD8α (53-6.7, dilution 1/400), anti-CD19 (1D3, dilution 1/400), anti-CD138 (281-2, dilution 1/200), anti-CD45.1 (A20, dilution 1/400), GL-7 (dilution 1/100), anti-CD95 (Jo2, dilution 1/100), anti-Bcl6 (K112-91, dilution 1/50) and anti-CD64 (X54-5/7.1, dilution 1/200). The following monoclonal antibodies purchased from eBioscience were used: anti-B220 (RA3-6B2, dilution 1/200), anti-CD4 (RM4-5, dilution 1/400), anti-PD-1 (J43, dilution 1/200), anti-Foxp3 (FJK-16 s, dilution 1/100), anti-CD44 (IM7, dilution 1/800) and anti-CD11c (N418, dilution 1/200). Anti-Neuropilin-1 (761705, dilution 1/400) was from R&D. Anti-PD-L1 (10F.9G2, dilution 1/200) was from Biolegend. For intracellular staining, cell suspensions were fixed and permeabilized using the BD Fixation/Permeabilization kit (Cat#555028). Before permeabilization, cells were stained with Fixable Viability Dye eFluor506 (eBioscience, Cat#65-0866). Labelled cells were acquired and analysed using an LSRII flow cytometer (BD Biosciences, San Jose, CA) and FlowJo software (Tree Star, Ashland, OR). Doublets and dead cells were excluded using appropriate FSC/SSC gates.

### ELISA

MOG-specific IgG was detected in the plasma from blood with ELISA using the SensoLyte Anti-MOG(35-55) IgG Quantitative ELISA Kit (Anaspec, Cat# AS-54465) as described by the manufacturer.

### Statistical analysis

Differences between variables were evaluated using the non-parametric Mann–Whitney test. All statistical analyses were carried out with the Prism 4.0 software (GraphPad). *P* values less than 0.05 were considered statistically significant.

## Additional information

**How to cite this article:** Aloulou, M. *et al*. Follicular regulatory T cells can be specific for the immunizing antigen and derive from naive T cells. *Nat. Commun.* 7:10579 doi: 10.1038/ncomms10579 (2016).

## Supplementary Material

Supplementary Information Supplementary Figures 1-13
