# Supplementary material for: Follicular regulatory T cells can be specific for the immunizing antigen and derive from naive T cells
Source: Nat Commun. 2016 Jan 28;7:10579. doi: 10.1038/ncomms10579 (PMC4738360; doi:10.1038/ncomms10579)
Supplement: Supplementary Information — Supplementary Figures 1-13 [file ncomms10579-s1.pdf]

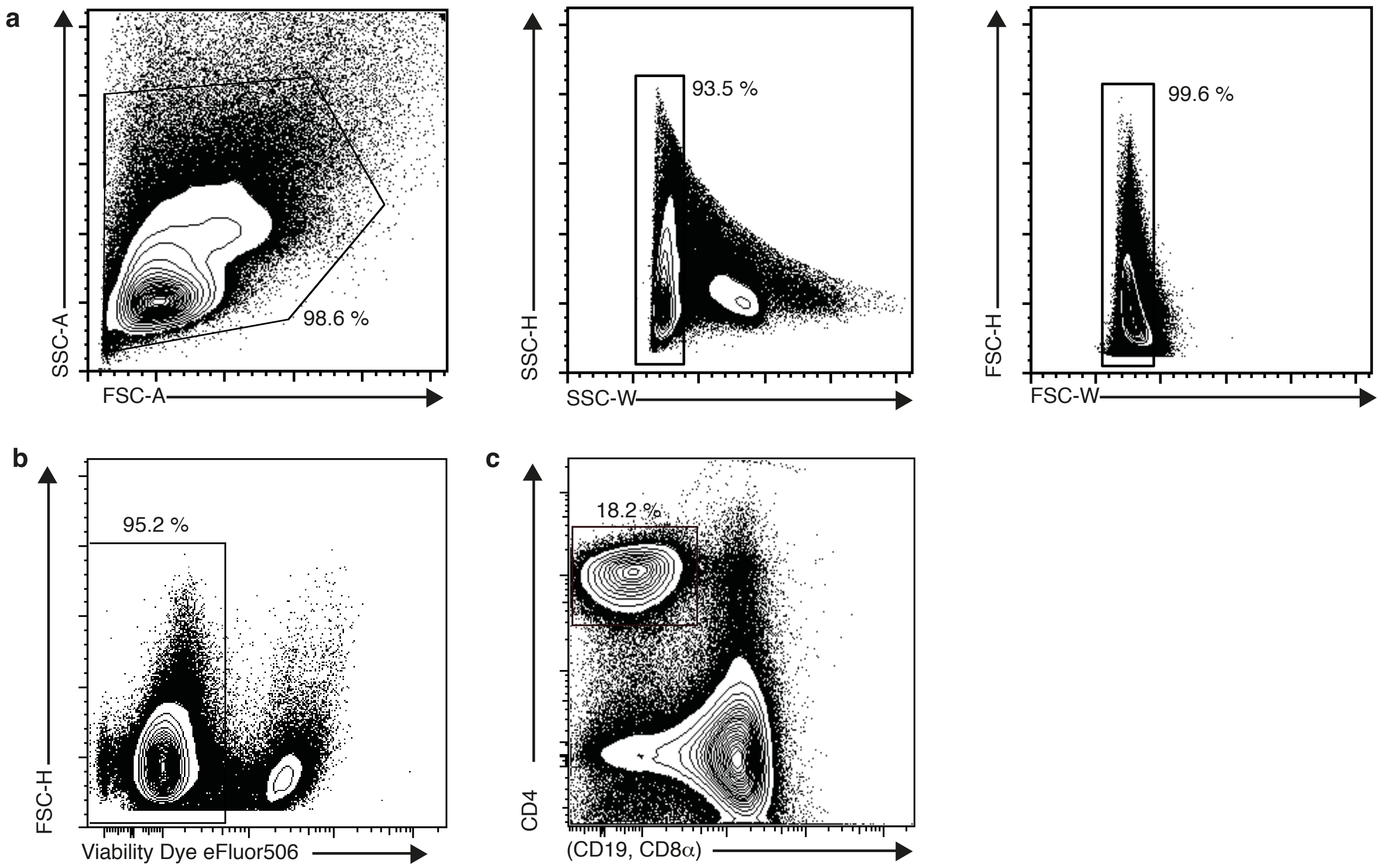

Supplementary Figure 1
Gating strategy to get to CD4+ cells by flow cytometry
WT mouse 7 days after immunization with MOG35-55 emulsified in CFA. dLN were harvested for the detection of cells. Doublets cells were excluded using appropriate FSC/SSC gates (a) as well as dead using Viability dye (b). Then CD4+ (CD19, CD8a)- were gated. Data shown are from a single experiment with one mouse and are representative of five independent experiments.

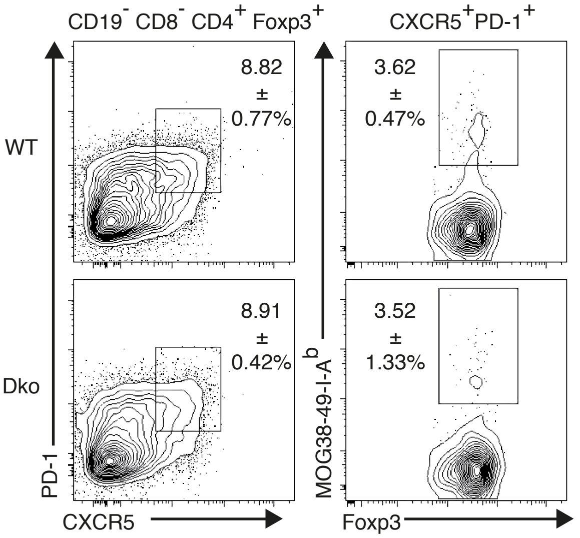

Supplementary Figure 2

CXCR5 and PD-1 stainings at the surface of CD19- CD8- CD4+ Foxp3+ cells from WT and Dko mice 7 days after immunization with MOG in CFA.
MOG38-49-I-Ab and Foxp3 stainings at the surface of CXCR5+PD-1+ cells.
The numbers in the dot plots represent the mean±SEM.
Data shown are from a single experiment with 5 mice and are representative of three independent experiments.

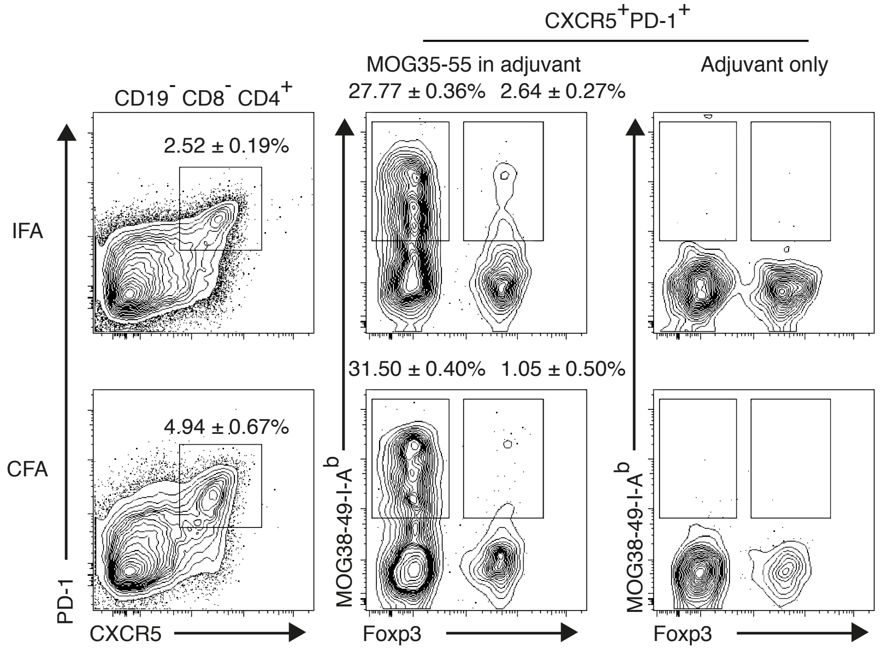

Supplementary Figure 3

CXCR5 and PD-1 stainings at the surface of CD19- CD8- CD4+ cells from Dko mice 7 days after immunization with MOG in IFA or CFA. MOG38-49-I-Ab and Foxp3 stainings at the surface of CXCR5+PD-1+ cells. The numbers in the dot plots represent the mean±SEM. Data shown are from a single experiment with 5 mice and are representative of three independent experiments.

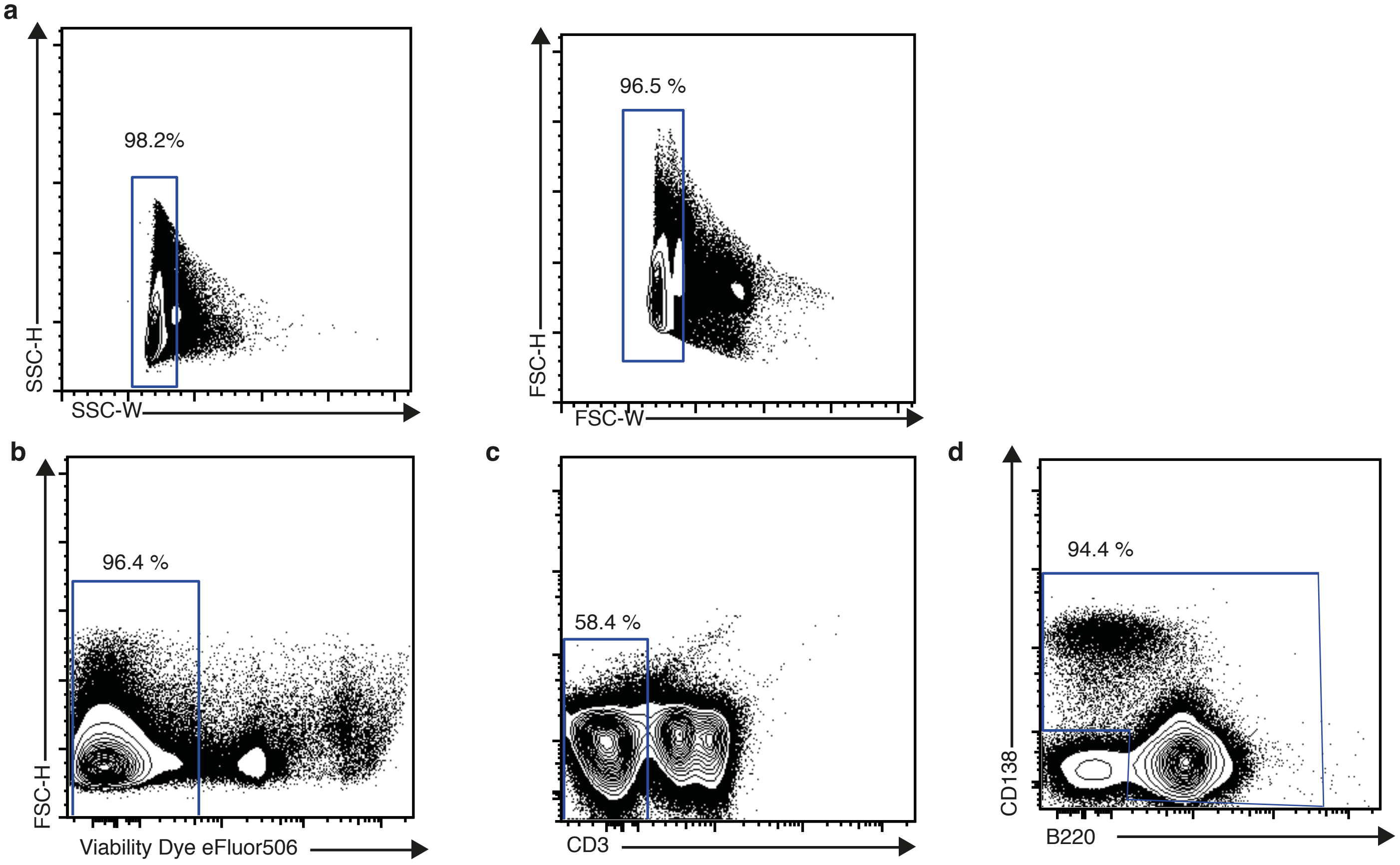

Supplementary Figure 4
Gating strategy to get to B cells by flow cytometry
WT mouse 12 days after immunization with MOG emulsified in CFA. dLN were harvested for the detection of cells. Doublets cells were excluded using appropriate FSC/SSC gates (a) as well as dead using Viability dye (b). Then B cells were gated selected as CD3- (c) and CD138+ or B220+ (d).
Data shown are from one mouse and are representative of five independent experiments.

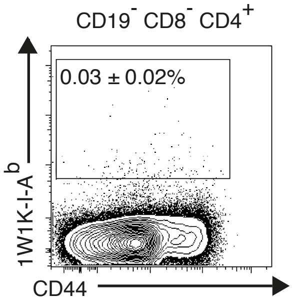

Supplementary Figure 5
Periaortic and inguinal LN were collected from unimmunized C57Bl/6 (WT) mice, 1W1K-I-Ab tetramer and CD44 stainings are depicted.
The numbers in the dot plot represent the mean±SEM.
Data shown are from a single experiment with 5 mice and are representative of five independent experiments.

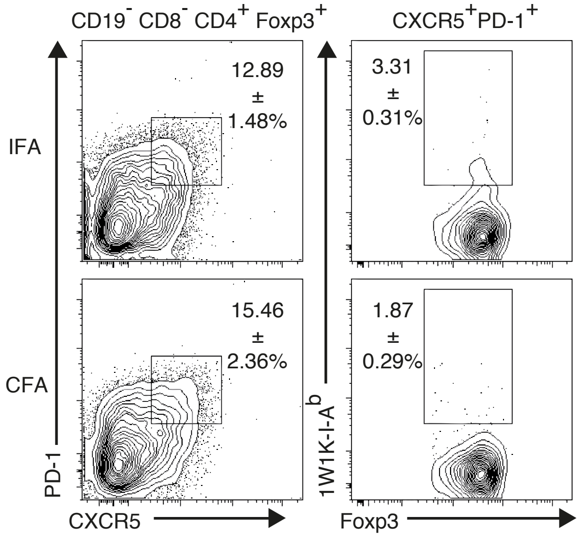

Supplementary Figure 6

WT mice immunized with 1W1K emulsified in IFA or CFA.
dLN were harvested for the detection of CXCR5 and PD-1 among CD19- CD8- CD4+ Foxp3+ cells and for 1W1K-I-Ab and Foxp3 at the surface of CXCR5+ PD-1+ cells.
Data are from one experiment of 6 mice per strain and representative of three independent experiments.

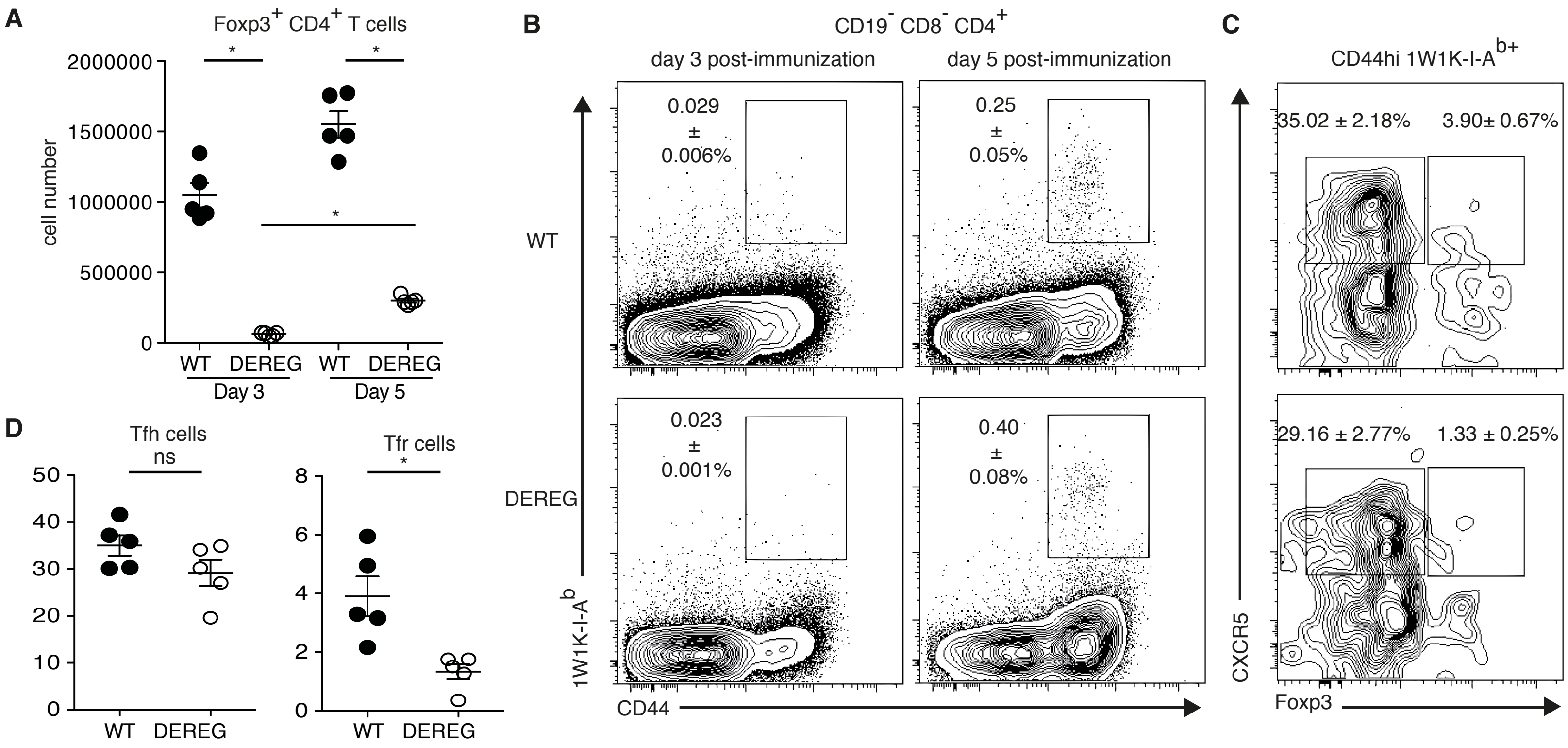

Supplementary Figure 7

Induction of Tfr cells in the first 48hours after immunization

DEREG and WT mice were immunized with 1W1K emulsified in IFA and treated with DTx at day 0 and day+1.

a) Absolute numbers of total Foxp3+ CD4+ T cells 3 and 5 days after immunization

b) 1W1K-IAb tetramer and CD44 staining of C19- CD8- CD4+ cells at day 3 and day 5 post-immunization

c) CXCR5 and Foxp3 staining at the surface of day 5 1W1K-specific Th cells

d) Frequencies of Tfh and Tfr among 1W1K-specific Th cells in dLN 5 days after immunization in WT and DEREG mice.

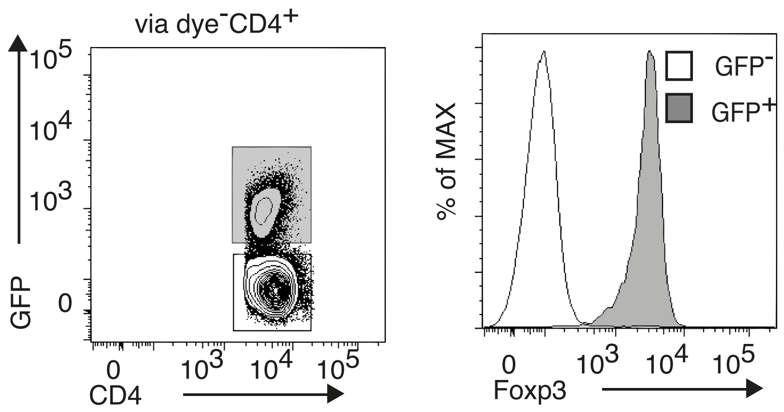

Supplementary Figure 8

GFP expression in CD4+ T cells from DEREG mice perfectly correlates with Foxp3 expression

CD4 + T cells from DEREG mice were collected and GFP and Foxp3 stainings were tested.

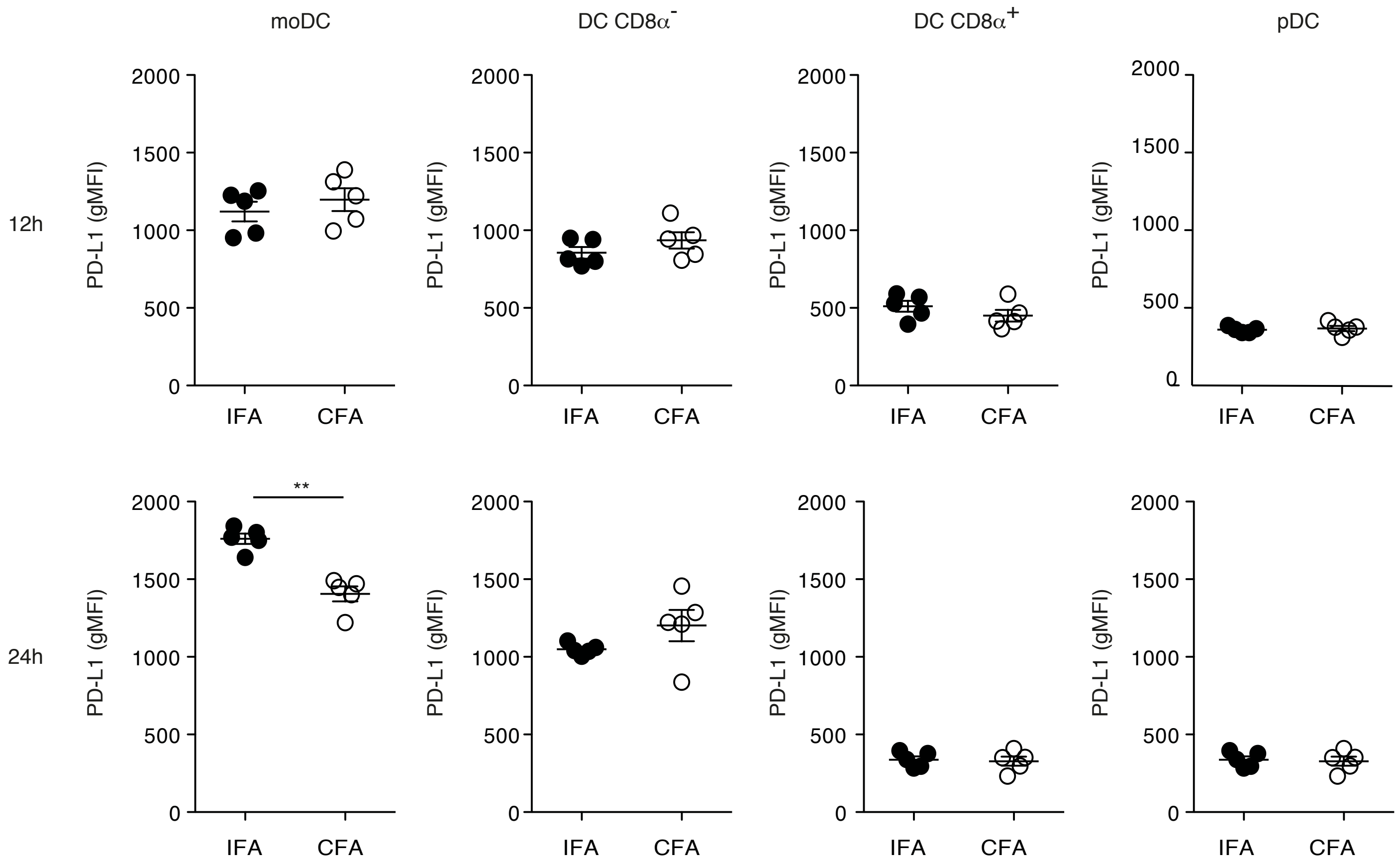

Supplementary Figure 9
PD-L1 expression at the surface of DC after immunization
gMFI of PD-L1 expression at the surface of conventional CD8α- DC, moDC, CD8α+ DC and plasmacytoid DC in the dLN 12 and 24 hours post-immunization of WT mice with Ea-OVA in IFA or CFA.

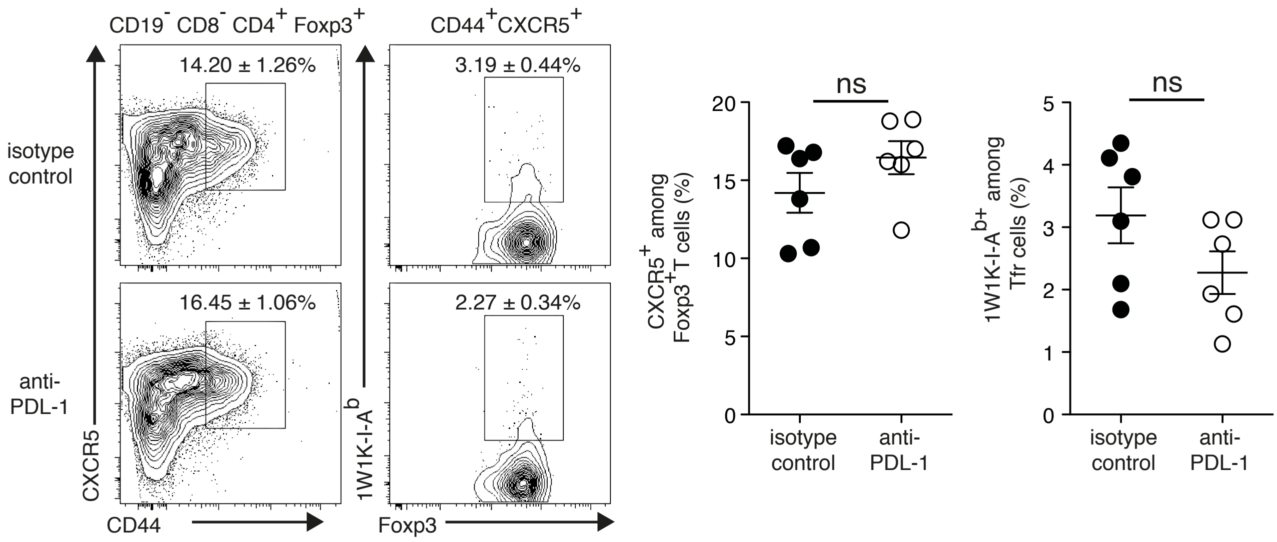

Supplementary Figure 10

Flow cytometric dot plots showing CXCR5 and CD44 stainings of CD19- CD8- CD4+ Foxp3+ cells and of 1W1K-IAb and Foxp3 stainings of CD44+ CXCR5+ cells in the dLN 7 days post-immunization with 1W1K in IFA and treated with anti-PD-L1 (white circles) or isotype control (black circles) at day 0 and day+2.

Data shown are from one experiment with 6 mice and are representative of two independent experiments.

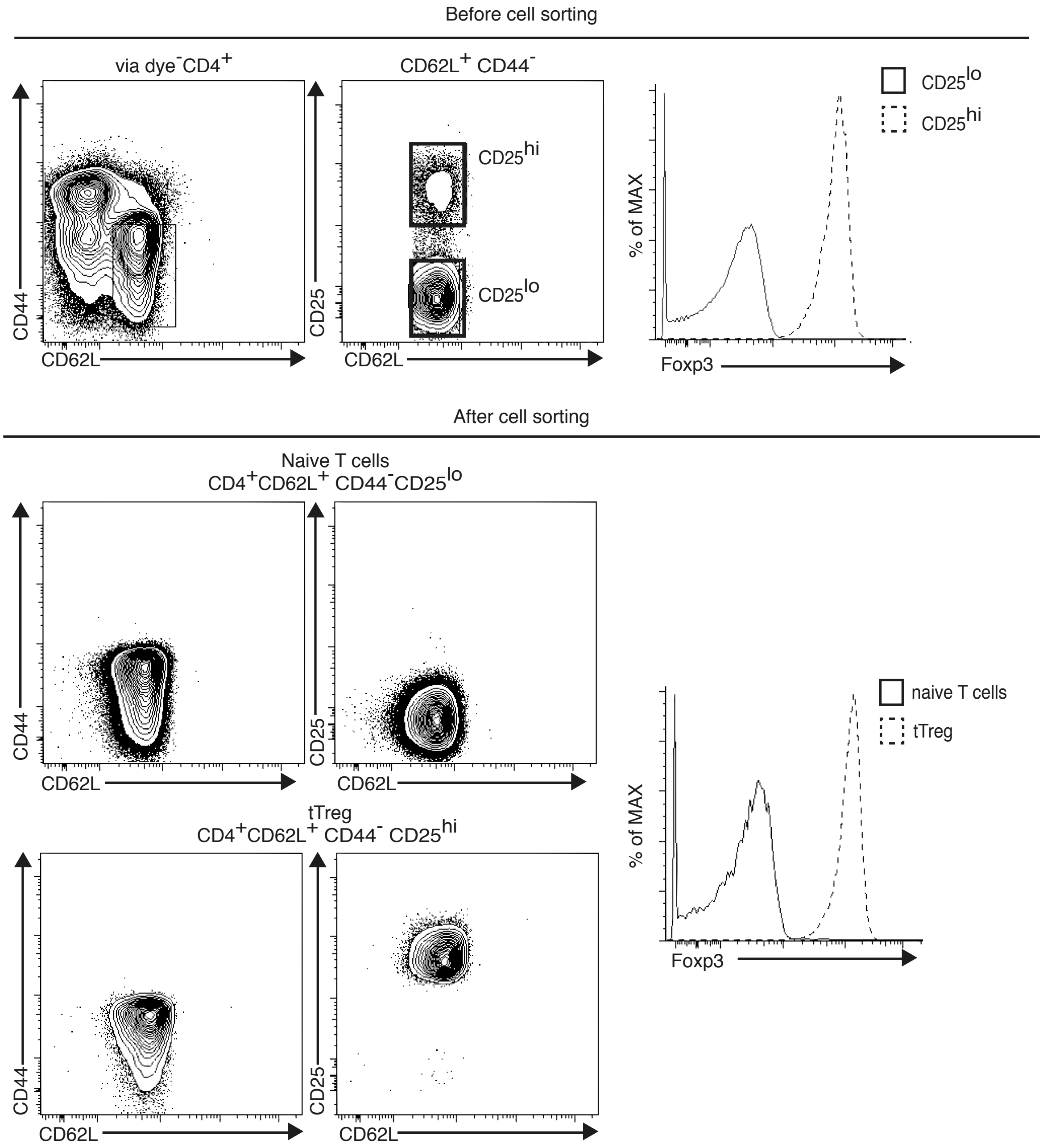

Supplementary Figure 11

Strategy of cell sorting of Naive CD4+ T cells

Spleen and LN from CD45.1 wild-type mice were collected and naive CD4+ T cells and tTreg were sorted out by flow cytometry as depicted.

Foxp3 expression is also shown before and after sort and demonstrates that all naive CD4+ T cells are Foxp3- as compared to tTreg that are all Foxp3+

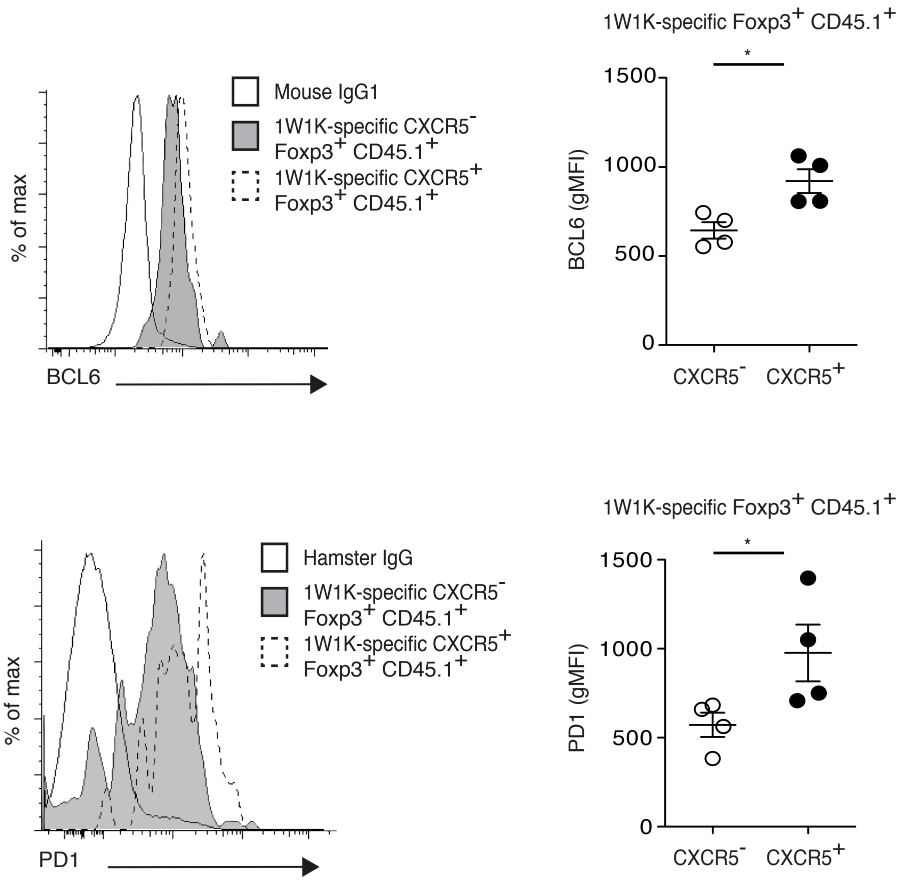

Supplementary Figure 12

PD-1 and Bcl6 expression of induced 1W1K-specific Foxp3+ Th cells

PD-1 and Bcl6 expression in transferred 1W1K-specific CD4+ FoxP3+ T cells (CD44+ 1W1K-IAb+ CD45.1+) 7 days after immunization with 1W1K in IFA

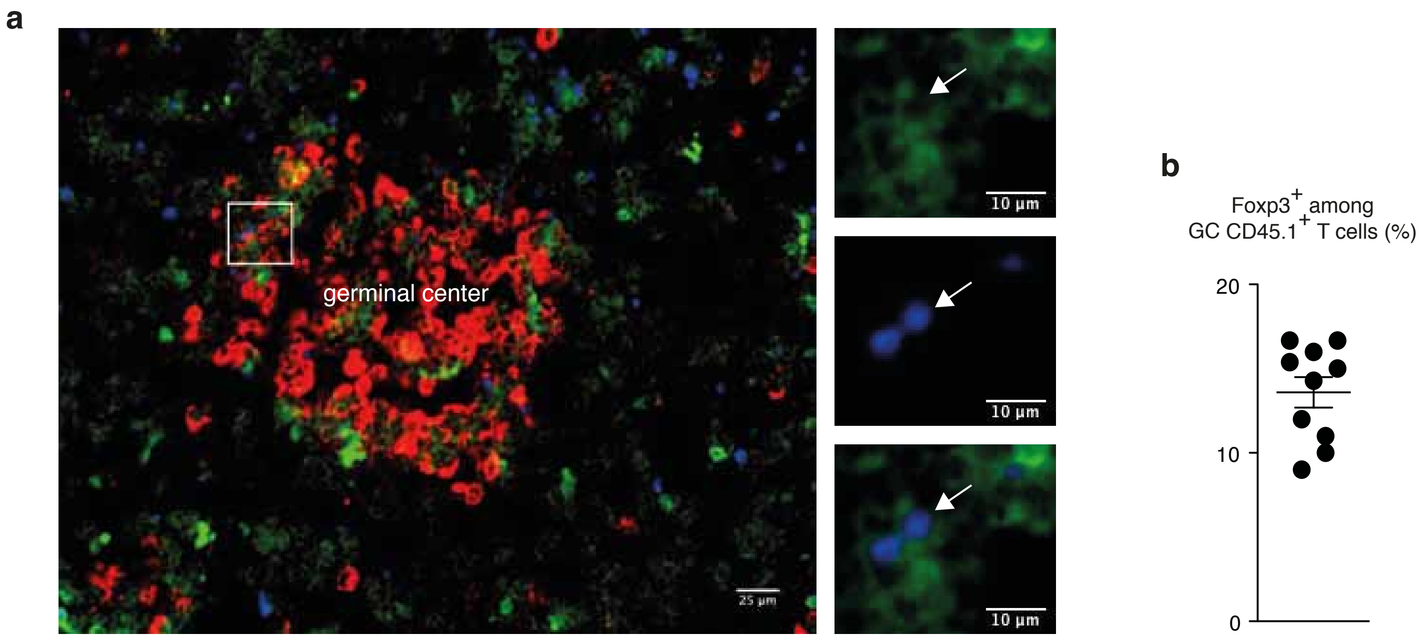

Supplementary Figure 13
Induced Tfr localize to the GC

Naïve CD4+ CD45.1+ T cells were injected iv into CXCR5-deficient T chimeras that were sc immunized the day after with 1W1K in IFA. Seven days after, confocal microscopy studies of dLN were performed using anti-Foxp3 (blue), anti-CD45.1 (green) and GL-7 (red) mAb.
dLN were harvested into PLP buffer (0.05M phosphate buffer containing 0.2ml-lysine [pH 7.4], 2 mg/ml NaIO4, 10 mg/ml paraformaldehyde), fixed overnight and dehydrated in 30% sucrose prior to embedding in OCT freezing media (Sakura Fineteck).
Frozen sections were cut on a CM1950 Cryostat.
Sections were stained in PBS/0.01% Triton X-100/5% goat serum.
Images were acquired on a Apotome ZEISS Inv. Localization of FoxP3+CD45.1+ cells in GC (GL-7+) is highlighted (scale bars; 25 μm, 10 μm for inset)(**a**).
Percentages of FoxP3+ cells among CD45.1+ cells in GC were qantified from 10 representative GCfrom 4 different mice (**b**).
